# 3D Imaging of Proton FLASH Radiation Using a Multi-Detector Small Animal PET System

**DOI:** 10.3390/tomography11120131

**Published:** 2025-11-26

**Authors:** Wen Li, Yuncheng Zhong, Youfang Lai, Lingshu Yin, Daniel Sforza, Devin Miles, Heng Li, Xun Jia

**Affiliations:** Department of Radiation Oncology and Molecular Radiation Sciences, Johns Hopkins University, Baltimore, MD 21287, USA

**Keywords:** PET, proton therapy, FLASH

## Abstract

**Objectives:** Ultra-high dose-rate FLASH radiotherapy has demonstrated strong potential in reducing normal tissue toxicity while maintaining effective tumor control. However, its underlying radiobiological mechanisms remain unclear, highlighting the need for novel approaches to probe the effects of radiation during and immediately after delivery. This study presents the first exploration of 3D PET imaging of positron-emitting nuclei (PENs) generated by a FLASH proton beam. **Methods:** A home-built 12-panel preclinical small-animal PET system was employed for recording coincidence events. A 142.4 MeV FLASH proton beam with a 100 ms delivery time was directed into a solid water phantom. PET coincidence signals were recorded during the first 1 s and up to 11 min. The system’s capability for 3D localization was also assessed, and Monte Carlo simulations were performed for validation. **Results:** The PET system successfully recorded coincidence data within the first second, including the 100 ms beam delivery interval. Detector dead-time effects under the high beam flux were observed, leading to underestimated event counts. Following irradiation, the measured activity and decay behavior were consistent with simulations. The PET system accurately reconstructed the spatial distribution of PEN activities, with discrepancies in measured versus calculated line profiles ranging from 3.35–6.85%. Reconstructed PET images enabled reliable 3D localization with sub-millimeter accuracy in both lateral and depth dimensions. **Conclusions:** Our findings demonstrate that a multi-detector PET system is a promising tool for investigating the radiation effects of FLASH beams.

## Simple Summary

Ultra-high dose-rate FLASH radiotherapy has emerged as a promising paradigm in radiation oncology. However, its physical and radiobiological mechanisms remain incompletely understood. In this study, we investigated a PET system equipped with 12 detector panels to analyze the distribution of PET events during and immediately after irradiation of a proton FLASH beam, validate these events using Monte Carlo simulations, and assess the system’s 3D localization performance. The system demonstrated the ability to reliably capture PET signals both during and after beam delivery, enabling accurate coincidence detection and having the potential to support real-time monitoring of proton FLASH beam-induced positron-emitting nuclei.

## 1. Introduction

Ultra-high dose-rate FLASH radiotherapy (RT) is an emerging technique recognized as one of the most significant breakthroughs in radiation oncology [1,2]. FLASH RT delivers a high dose of radiation to patients in an ultra-short time frame (generally ≥ 40 Gy/s and in less than 200 ms [2]), enabling tumor control comparable to conventional RT while significantly reducing toxicity to surrounding normal tissues [3]. This phenomenon, known as the FLASH effect, was first reported by V. Favaudon et al. in 2014, who demonstrated that a single FLASH treatment of 17 Gy could significantly inhibit the onset and progression of pneumonia and pulmonary damage while maintaining effective tumor control [4]. In 2019, the first human FLASH treatment was successfully administered to a patient with multi-resistant CD30+ T-cell cutaneous lymphoma. After a 5-month follow-up, the treatment showed promising outcomes in both skin protection and tumor control, demonstrating its potential clinical feasibility [1]. More recently, E. Mascia et al. conducted a clinical trial involving 10 patients (aged 27–81 years) with symptomatic bone metastases [5]. Their study found that treatment efficacy and adverse event profiles were comparable to those of standard-of-care RT, further confirming the clinical feasibility of FLASH RT. In addition to its normal tissue protection benefits, FLASH RT also offers potential economic advantages by substantially reducing treatment time and fractionation, thereby increasing patient throughput in hospitals.

Despite these promising findings, FLASH RT remains in the experimental and early clinical trial phases. The physical and radiobiological mechanisms underlying the FLASH effect are still not fully understood, posing a major challenge for clinical translation. It is therefore critical to investigate the radiation physics and chemistry effects triggered by the ultra-high dose-rate delivery, as these may play key roles in initiating the unique biological responses associated with FLASH effect. A thorough understanding of these effects is essential for elucidating the underlying mechanism, optimizing treatment parameters, and safely translating FLASH RT into routine clinical practice.

Methods for probing physical effects during and immediately after FLASH delivery target the sub-second or shorter time windows. Existing representative studies are summarized in Table 1. Time-resolved dosimetry uses chambers corrected for recombination and diamond/plastic-scintillator detectors to characterize dose-per-pulse and instantaneous dose rate under FLASH conditions [6,7]. Optical methods, such as Cherenkov emission imaging, provide real-time, pulse-to-pulse visualization and output tracking during ultra-high dose-rate electron FLASH RT, including the first in vivo demonstrations [8,9]. Complementary fast-scintillator systems enable prompt light readout for dose-rate monitoring [10]. Prompt-gamma and related secondary-radiation signatures are also being explored as surrogates for characterizing the temporal structure of FLASH beams [11]. Beyond physics-based effects, ultrafast radiolysis and oxygen dynamics are investigated through both computational and experimental approaches, including studies of radiolytic oxygen depletion, quantitative analyses of oxygen tension, and real-time optical oximetry during FLASH irradiation [12,13,14,15].

During FLASH beam delivery, positron-emitting nucleus (PENs) generated by protons (e.g., ^11^C, ^15^O, and ^13^N) emit strong, distinct signals that persist throughout the spill duration and after, providing a unique opportunity to capture a snapshot of the delivered dose using a real-time positron emission tomography (PET) system. PET imaging can non-invasively track these isotopes, thereby verifying beam delivery and providing quantitative data that reduce uncertainties. Beyond dose verification, PET offers critical insights into the underlying physics and biological processes in FLASH RT by enabling correlation of isotope distribution patterns with dose-rate effects, tissue composition, and potential radiochemical changes induced by ultra-high dose-rate irradiation.

A recent study reported that a PET system with two detector modules could successfully record activations in a cylindrical polymethyl methacrylate phantom irradiated by a FLASH proton beam at a dose rate of 164 Gy/s over a 101.5 ms spill [16]. In a subsequent study, the same group extended their work to measure both in-spill gamma and post-spill emissions (up to 20 min), further validating the potential of PET for imaging and monitoring FLASH proton therapy [17]. However, these studies used only two detector modules, which resulted in low sensitivity due to the limited data acquisition geometry. Moreover, the incomplete geometry prohibited image reconstruction for deriving spatial information induced by the FLASH beam.

Motivated by the strong potential of in-beam PET imaging for FLASH proton therapy, this study investigates a PET system with a complete ring consisting of 12 detector panels, enabling 3D PET imaging of PENs under FLASH irradiation. Our PET system provides comprehensive coverage of the imaging area, allowing for more accurate and detailed recordings of FLASH proton beam events with improved sensitivity. The enhanced accuracy and reliability of the recorded data support better imaging and monitoring of the FLASH proton beam during and after delivery, ultimately advancing the development of PET-guided FLASH RT. Furthermore, the spatial imaging information provided by our PET system enables 3D beam localization [18,19], which is critical for confirming that the proton beam follows the planned trajectory and deposits dose at the intended depth. This is particularly important in FLASH RT, where the ultra-high dose-rate deposition leaves virtually little to no margin for error.

## 2. Methods

### 2.1. Experimental Setup

**Figure 1:**
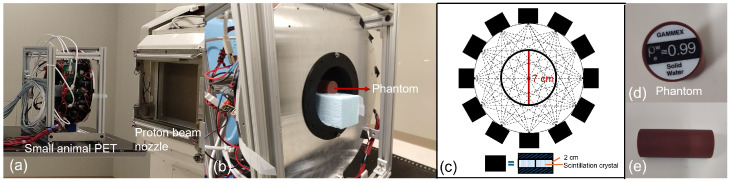
Experimental setup. (**a**) Illustration of the proton beam nozzle and the small-animal PET; (**b**) Front view of the PET system with a solid water phantom; (**c**) PET geometry with a cross-axial FOV diameter of 7 cm; (**d**,**e**) Top and side views of the solid water high-equivalency phantom used in this study.

Experiments were performed at the Johns Hopkins Proton Therapy Center, Sibley Memorial Hospital, Washington, DC, USA. The setup consisted of three primary components: a multi-panel PET system, the FLASH proton beam, and a solid water phantom (Figure 1).

#### 2.1.1. PET Imaging System

A home-built small-animal PET system dedicated to preclinical research was utilized in this study (Figure 1a–c). The PET system consists of 12 detector panels arranged in a dodecagonal configuration [20,21]. The distance from the front surface of each panel to the center is 7.0 cm. Each panel contains four closely tiled detectors, and each detector comprises a 15 × 15 array of LYSO scintillation crystals. The size of crystals is 1 × 1 × 20 mm^3^. The end of each crystal is optically coupled to an 8 × 8 array of silicon photomultiplier arrays (HAMAMATSU Photonics K.K., model S13361-2050-08) with an active pixel size of 2 × 2 mm^2^ and depth-of-interaction (DOI) measurements. All sides of the scintillator are surface-ground with 0.03 mm lapping to balance detector performance. These panels form 42 detector pairs within the field of view (FOV). The overall size of the detector head is 33 cm in diameter and 11 cm in axial length, with a cross-axial and axial FOV diameter of 7 cm and a length of ∼3.3 cm, respectively. The coincidence timing resolution of the detector panel pair is approximately 3.6 ns. The output singles event information includes event energy, interaction location, and arrival timing.

The coincidence processor is a key component in this PET system, which selects valid coincidences from gamma interactions to reconstruct the image of PEN distribution. The conventional centralized coincidence processor (CCP) has limitations in processing data from a large number of detector pairs with ultra-high data throughput due to its single-thread sequential processing nature. In the multi-panel PET system in our study, a set of distributed coincidence processors (DCP) with 42 processors is used to select valid coincidence events. Each processor selects coincidence events for one specific detector pair only. Different processors work in parallel to accelerate the computation process. Compared with CCP, the DCP minimizes data processing delay and improves coincidence event counting rates, benefiting ultra-high data throughput scenarios and enhancing imaging performance. This is particularly useful for 3D imaging which utilizes a multi-panel system with high data throughput in our study. For each detector panel pair, the maximum detected singles event rate is 2.5 × 10^5^/s. With 42 panel pairs in total, the maximum system coincidence event processing rate is 1.05 × 10^7^/s due to the parallel processing.

The system electronics are developed with off-the-shelf components. Different detector panels are connected to the system electronics board using mini-DP cables, which enables the placement of system-level electronics outside the proton beam nozzle. Each mini-DP cable includes three differential signal pairs: two downstream pairs (providing a 50 MHz clock and a system electronics-detector synchronization signal) and one upstream pair (providing 400 Mbps low-voltage differential signals for detector-system electronics data transmission). The output coincidence data is transferred to the computer via a PCI-e cable, and the collected data is formatted in list-mode.

#### 2.1.2. FLASH Proton Beam

A multi-room synchrotron system (Hitachi Probeat CR, Hitachi Ltd., Tokyo, Japan), designed with a compact gantry footprint specifically for proton pencil beam scanning, was employed in our experiments. The FLASH proton beamline was characterized at our institution by Lingshu et al. [22,23]. During FLASH proton beam delivery, the linear accelerator first accelerates protons to 7 MeV. These protons are then injected into the synchrotron’s acceleration ring, where they are further accelerated to 142.4 MeV before extraction for irradiation. The beam extraction radio-frequency (RF) wave pattern is optimized by the Hitachi engineering team to maximize the number of protons extracted per spill while minimizing beam output fluctuations. The FLASH proton spill lasts for 100 ms, with timing controlled by the simulated pulse signal interface (SPSI) in the Hitachi synchrotron system, which is set to a 10 kHz pulse frequency for the waveform generator. Due to the RF pattern design, the beam current peaks at ∼150 nA at the start to maximize proton numbers, then gradually decreases to ∼50 nA by the end of the 50 ms. The total number of protons extracted during this 100 ms for a 142.4 MeV FLASH beam is approximately 3.10 × 10^10^, yielding an average synchrotron current of 99.20 nA.

#### 2.1.3. Phantom

A solid water High Equivalency (HE) phantom (GAMMEX, Sun Nuclear Corporation, Melbourne, FL, USA) was utilized in this study. Solid Water phantom has been widely accepted as a gold standard for routine dosimetry quality assurance. The main components of the Solid Water phantom are epoxy resins to provide necessary structure integrity and durability, and various powders to control the density and radiation properties. The Solid Water HE has a water equivalence within 0.5% for therapeutic and diagnostic energy ranges. The mass density and electron density of the solid water HE are around 1.032 g/cm^3^ (±0.005 g/cm^3^) and 0.557 e^−^/cm^3^, respectively [24]. A cylindrical solid water HE phantom with diameter of 28 mm and length of 70 mm was used in this study for characterization of the FLASH proton beam, as shown in Figure 1d,e. The phantom was supported by a foam block, positioned at the center of the PET system’s FOV, and approximately aligned with the central axis of the beam (Figure 1c). Additionally, since the range of the 142.4 MeV proton beam is larger than the length of this phantom, we placed a ∼12.6 cm-thick solid water slab upstream to the phantom (not shown in Figure 1), so that the end region of the proton beam was within the PET FOV.

### 2.2. Study Design and Evaluation

The main studies included analyzing the time distribution of recorded PET events during and immediately after irradiation, validating the PET events using Monte Carlo (MC) simulations, and verifying the 3D localization performance of the multi-detector PET system.

#### 2.2.1. Time Distribution of PET Events

To record the PET events with our multi-panel PET system, we irradiated the solid water phantom using the FLASH proton beam. The data acquisition was performed for a total duration of 11 min, starting at the onset of beam delivery and continuing throughout and after the irradiation.

For the events recorded, we performed three procedures to refine the PET coincidence event selection: removal of multiple coincidences, narrowing of the coincidence time window, and application of an energy window. In PET acquisition, more than two gamma photons may be detected within the same time window, resulting in multiple coincidences. These events are ambiguous, because it is unclear which photon pairs truly originate from the same annihilation event. Excluding such cases and retaining only valid two-photon events improves the accuracy of image reconstruction. In addition, we used a short coincidence time window of 10 ns. Since the time window defines the maximum delay between two detected photons to be considered coincident, a wide window increases the likelihood of random coincidences, in which unrelated photons are mistakenly paired. Narrowing the window to 10 ns therefore reduces random coincidences and improves the reliability of coincidence detection. Finally, we applied an energy window of 360–650 keV. Although the true annihilation photon energy is 511 keV, detector resolution and scattering can cause variation in the measured energy. A wide energy acceptance would include many scattered photons, which have lost part of their energy before detection. Applying an appropriate energy window ensures that most of the detected events represent true coincidences.

PENs produced by the proton beam include ^10^C ($T_{1/2}=19.3$ s), ^11^C ($T_{1/2}=1224$ s), ^12^N ($T_{1/2}=0.011$ s), ^13^N ($T_{1/2}=598$ s), ^14^O ($T_{1/2}=70.9$ s), and ^15^O ($T_{1/2}=122$ s), which are generated through nuclear collisions between the high-energy protons and the nuclei in the phantom. We first characterized the temporal distribution of these PENs for the FLASH irradiation, which may provide insight into the underlying nuclear interactions and assessing the feasibility of PET-based beam monitoring. As such, we recorded coincidence events covering a 1.0 s time window that included 100 ms of irradiation time and 900 ms post-beam irradiation immediately after beam delivery. Acquired coincidence events as a function of time were analyzed. PET data acquisition was further extended to a total of 11 min to record the coincidence events for PENs with relatively long half-lives.

#### 2.2.2. Monte Carlo Simulation

To validate the recorded PET data, we performed MC simulations for the interactions of the FLASH proton beam with the solid water phantom using GATE software (Version 9.2, International OpenGATE Collaboration, Clermont-Ferrand, France) coupled with the Geant4 toolkit (The Geant4 Collaboration, Version 11.0.0) [25,26,27,28]. The QGSP_BERT_HP_EMY reference physics list was employed to enable high-precision modeling of neutron and electromagnetic physics processes.

In the simulation, the FLASH proton beam was modeled as an axially symmetric pencil beam incident on a cylindrical solid water phantom with a diameter of 28 mm and a length of 160 mm along its central axis. The beam energy was 142.4 MeV with a Gaussian intensity profile of 7.0 mm in standard deviation. The solid water phantom was defined according to ICRU report 44 [29], with the material properties matching the chemical composition used in our experiment and a density of 1.032 g/cm^3^.

We recorded the number of PENs produced by the proton beam in a 3D voxel grid with a voxel size of 1×1×1 mm^3^. All PENs were included except ^12^N, as the extremely short half-life of ^12^N renders its contribution negligible for comparison with experimental measurements. For further analysis, the output isotopes were converted to activities as

(1) $$A_{i}\left(t\right)=A_{i}\left(0\right)e^{-\lambda_{i}t}=\lambda_{i}N_{i}\left(0\right)e^{-\lambda_{i}t},$$

where $A_{i}\left(t\right)$ is the total activity at time *t* for isotope *i*, $A_{i}\left(0\right)=\lambda_{i}N_{i}\left(0\right)$ is the initial activity at t=0, and $\lambda_{i}=\ln2/T_{1/2}^{\left(i\right)}$ is the decay constant derived from the isotope half-life $T_{1/2}^{\left(i\right)}$. Using these activities, activity–time curves were plotted for further analysis. To compare with the experiment results that recorded the number of coincident events in sequential one-minute time windows, each activity curve was integrated over the corresponding time window intervals. Normalization of the simulation results was applied to make the mean value of the simulation result equal to that of the measurements. After that, the simulation and experiment results were compared. Additionally, due to the capability of spatial imaging of the PET system, the simulated 3D distributions of activities were compared with the reconstructed PET images along the depth and lateral directions.

#### 2.2.3. 3D Localization with the Multi-Detector PET System

Compared to previous studies that used two-detector PET system to investigate the FLASH proton beam [16,17], our study employed a 12-panel PET system arranged in a dodecagon configuration, enabling 3D monitoring of FLASH beam interactions. To assess the 3D localization capability of the multi-detector PET system, we initially placed the phantom at the center of the FOV, with a 12.6 cm-thick solid water slab positioned upstream of the PET system and the phantom. The phantom was then irradiated using the 142.4 MeV FLASH proton beam (labeled as initial irradiation). After the initial irradiation, the couch was shifted laterally by 5 mm, and the thickness of the solid water slab was reduced by 6 mm to 12.0 cm for the irradiation with the same beam (labeled as second irradiation).

The recorded PET coincidence events from these two irradiation experiments were reconstructed into 3D PET images with a voxel size of 1×1×1 mm^3^ using an open-source CASToR software (Version: 3.1, CASToR Organization, Brest, France) [30] with the maximum likelihood expectation-maximization (MLEM) algorithm [31]. The reconstruction process was conducted with a stopping criteria of 9 iterations. This number of iterations was chosen to balance image contrast and background noise. To exclude background noise and focus more on the PET events for better image analysis, we created a cylinder region covering the beam direction, with a length of 36 mm and a diameter of 40 mm, larger than the phantom’s 28 mm diameter. Voxel values outside the cylinder were set to 0, while those inside the region remained unchanged.

With the reconstructed image, we first compared the reconstructed PET image with the MC simulated activity image for the first irradiation case. As such, intensity profiles in both the range and lateral directions were analyzed. It is worth mentioning that in our experiments, the PET system only covered 3.5 cm range in the depth direction, and a 12.6 cm thick solid water slab was placed upstream of the solid water phantom and the PET system. Hence, we only compared the simulated data with measurements in the range covered by the PET system.

Furthermore, both lateral and range direction movement were evaluated based on the reconstructed PET image. To assess the PET system’s ability to accurately capture lateral movement distances, we analyzed the reconstructed 3D PET images by summing PET image intensity along the range direction for both irradiation experiments. The peak position was identified in the resulting 2D images to represent the beam central locations. The shift between the central locations at the two irradiation experiments were calculated and compared with the expected value.

To evaluate the range shift caused by the reduced thickness of the upstream solid water slab, we plotted two line profiles of image intensity at the identified beam central location. The distance between the depths in the two irradiation experiments, where the PET image intensity reduced to 50% of the peak value was calculated to determine the range difference, which was then compared with the expected value.

## 3. Results

### 3.1. Characterization of PET Events During and Immediately After Irradiation

**Figure 2:**
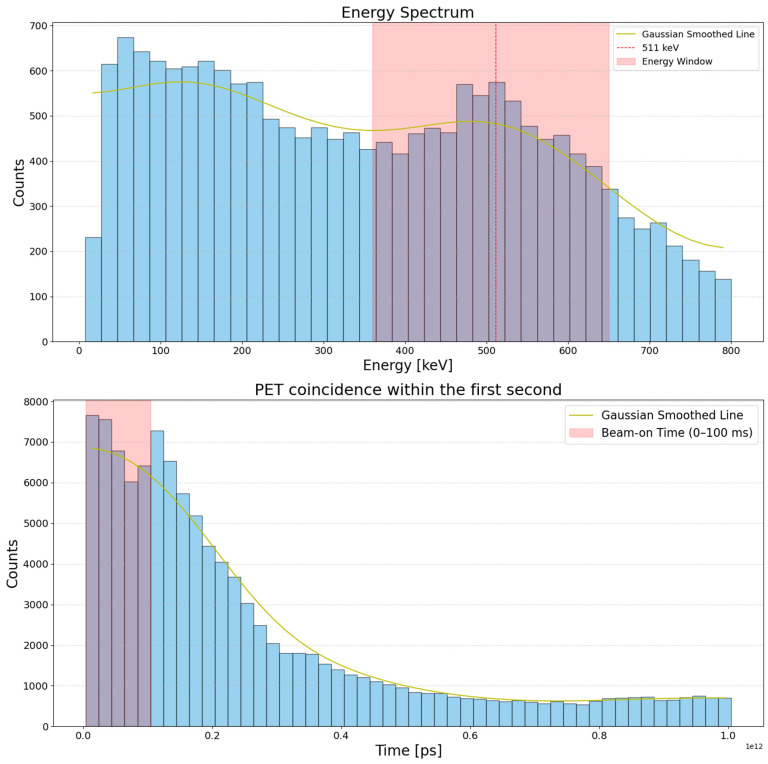
(**Top**): Measured energy spectrum. The histogram shows the experimental counts per energy bin. The vertical dashed line indicates the 511 keV photon peak position, corresponding to annihilation photons. The shaded area indicated the energy window used in our coincidence event selection. (**Bottom**): PET coincidence events within the first second. The beam-on time (first 100 ms) is marked in the shaded region.

To investigate the PET events during and immediately after beam delivery, we plot the energy spectrum and the recorded PET coincidences within the first second of irradiation, as illustrated in Figure 2. The energy spectrum was obtained by analyzing the energy in keV of event signals recorded at individual PET detectors. The data were binned into 40 bins with an upper cutoff at 800 keV. The spectrum clearly exhibited a clear peak at ∼511 keV, highlighting the capability of the PET system in capturing the positron annihilation events.

The lower panel of Figure 2 shows the temporal distribution of coincidence events, with the beam on time indicated by the shaded region. The data were binned into 50 bins with a bin width of 20 ms. During the beam delivery, there was clearly a burst of PEN production, as indicated by the substantial amount of PET coincidence events detected. We noticed a coincidence drop during the beam-on time. This might be caused by the saturation of the in-spill events recording. Due to the ultra-high dose rate of the FLASH beam, which generates an extremely large number of coincidence events in a very short time frame, the PET system’s detector and readout electronics may not be able to process these high-frequency events in real time, leading to a “dead-time” effect. Despite using the DCP with a maximum single-event detection rate of 2.5 × 10^5^/s, the extremely high event rates during the 100 ms FLASH beam extraction time still overwhelmed the system. Future improvements to the PET system, such as optimizing detector design, enhancing data processing capabilities, and developing more efficient coincidence processing algorithms, could help mitigate this issue.

Immediately after beam delivery, there was a rapid decay in counts over time. This decay behavior was the combined effect of several PENs with short half-lives, such as ^12^N and ^10^C. The quantitative interpretation of this early decay, however, is subject to several uncertainties. First, detector dead-time during and immediately after irradiation can lead to underestimated early PET counts, while substantial photon backgrounds due to prompt-gamma interactions and other radiation processes can further distort the signal. Second, detector temporal limitations, including finite timing resolution, pulse pile-up, and signal integration over nonzero time bins, can broaden the apparent fast-decaying component. Third, the pulsed temporal structure of the FLASH proton beam introduces additional convolution between isotope production and detection, further smoothing the measured decay profile.

### 3.2. Characterization of PET Events Post Irradiation

**Figure 3:**
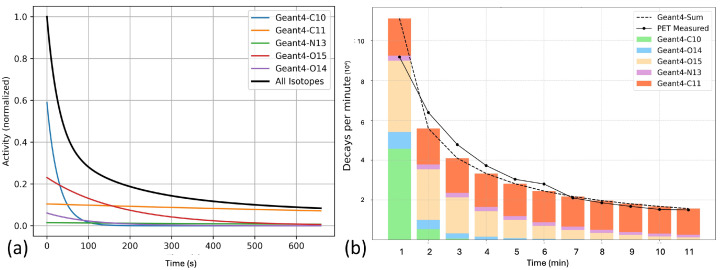
Time distribution of PET coincidence events post irradiation. (**a**) Simulated time-activity curve for all PENs; (**b**) Comparison between recorded number of PET events in each one-minute window and corresponding simulated results.

Figure 3a shows the time-activity curves of PENs during the 11 min time period derived by MC simulations. The initial numbers of these PENs were computed using MC simulations, and their corresponding activity curves were calculated using Equation (1). Among these isotopes, the contribution of ^10^C decreased rapidly within the first few minutes due to its short half-life ($T_{1/2}=19.3$ s). After about 150 s, ^11^C became the dominant component. The contribution of ^13^N and ^14^O to the total activity were relatively minor due to their low production yield.

As expected, a clear decay over time was observed experimentally, as shown by the black solid curve in Figure 3b, which reports the number of coincidence events in each one-minute interval. The stacked bars represent the simulated contributions from individual PENs within each interval, and the dashed line indicates their total value. The measured and simulated data showed reasonable agreement, confirming that the recorded PET signals reflect the expected isotope decay dynamics. The simulation slightly overestimated the measured counts in the first minute, likely due to missed detections in the experiment during the initial high-flux period, potentially caused by detector dead time or related effects.

### 3.3. PET Image Reconstruction

**Figure 4:**
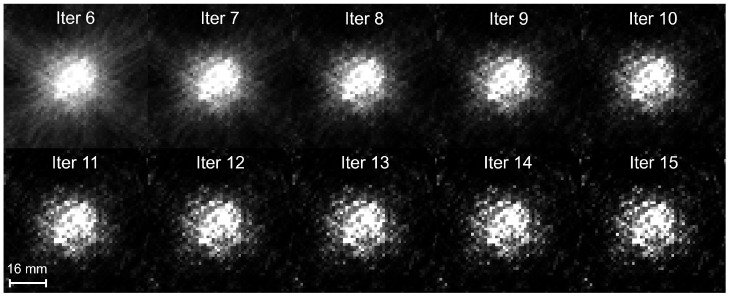
Reconstruction results at 10 different iterations.

Figure 4 shows one 2D image of the reconstructed PET image at 10 iterations (iterations 6–15). The 2D image was taken in a plane perpendicular to the beam at a depth of 6 mm. Based on these results, a stopping criterion of 9 iteration steps for the MLEM reconstruction algorithm was chosen to balance the image contrast and background noise.

**Figure 5:**
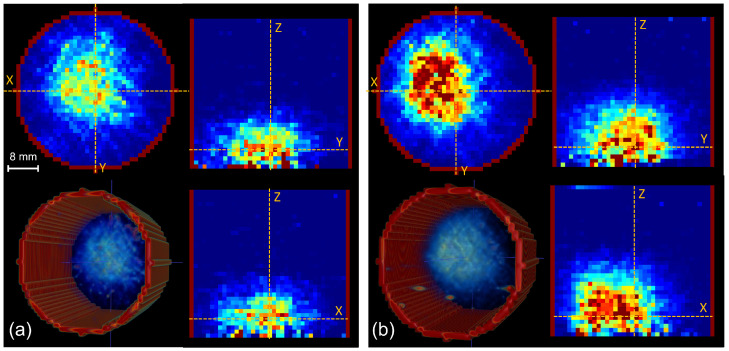
Reconstructed 3D PET images from the two irradiation experiments. (**a**) The reconstructed image of the initial irradiation; (**b**) The reconstructed image of the second irradiation after a 5 mm lateral couch shift and a 6 mm reduction in solid water slab thickness. Each sub-image displays: axial view (**top left**), sagittal view (**top right**), 3D view (**bottom left**), as well as coronal view (**bottom right**). Dashed lines in the three orthogonal views indicate locations of other views. X: Left–Right, Y: Anterior–Posterior, Z: Superior–Inferior).

**Figure 6:**
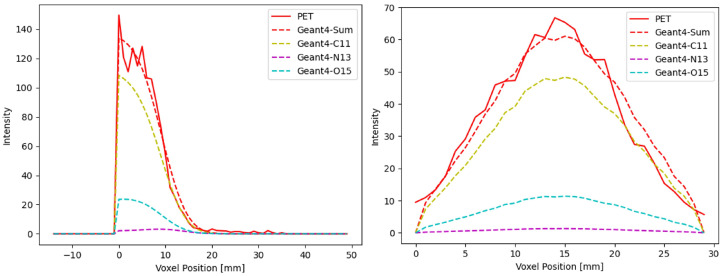
Comparison of measured PET image intensity with MC simulated results along the depth direction (**Left**) and lateral direction (**Right**).

The reconstructed 3D PET images in the two irradiation experiments are shown in Figure 5. The red cylinder indicates the region of interest manually defined during image reconstruction, outside of which the image intensity was set to zero to eliminate peripheral background noise. Image intensity profiles in both the depth and lateral directions of the first irradiation were extracted from the reconstructed 3D PET images (Figure 5a), as shown in Figure 6. The depth profile was at the beam central axis and the lateral profile was extracted at the depth of 5 mm. Both profiles were compared with corresponding MC simulation results.

In both the depth and the lateral directions, the PET-recorded activity profiles showed good agreement with the simulated distributions, confirming the PET system’s capability to accurately capture the spatial characteristics of activities induced by the FLASH proton beam. To quantitatively assess this agreement, we calculated the relative mean square error (rMSE) between the simulated and measured results, along with the percentage discrepancy based on the intensity range. The percentage discrepancies were 3.35% in the depth direction and 6.85% in the lateral direction, indicating a reasonable level of consistency between the PET measurements and the simulations. The discrepancy between the simulated and measured results can be attributed to the intrinsic limitations of the PET imaging system. While the simulation reflects only the physical isotope distribution, PET measurements are affected by multiple factors such as detector resolution, photon scatter, reconstruction algorithms, etc., all of which may contribute to the profile discrepancy. A possible solution would be to perform a full-system MC simulation that models the PET detector response, which could enable a more direct and accurate comparison between simulated and experimental results.

### 3.4. 3D Localization Capability Evaluation

**Figure 7:**
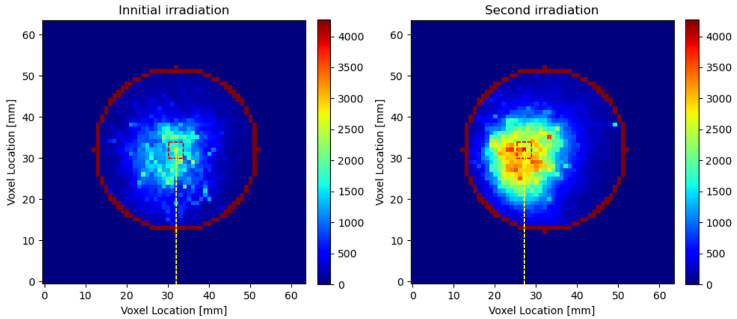
3D localization results in the lateral direction. The (**left**) panel shows the voxel value summation along the depth direction, with the beam center identified at (32, 32) mm in the PET image for the initial irradiation. The (**right**) panel presents the corresponding results after a 5 mm couch shift and a 6 mm reduction in Solid Water slab thickness, with the beam center identified at (27, 32) mm.

Figure 7 presents the images after summing along the 3D PET image intensity along the depth direction for the two irradiation experiments in Figure 5. With these two images, the beam centers were identified to be at (32, 32) mm and (27, 32) mm for the initial irradiation and the second irradiation, respectively. Thus, the identified beam center shift between the two irradiation experiments was (5, 0) mm. This result aligns well with the lateral couch movement distance of 5 mm.

**Figure 8:**
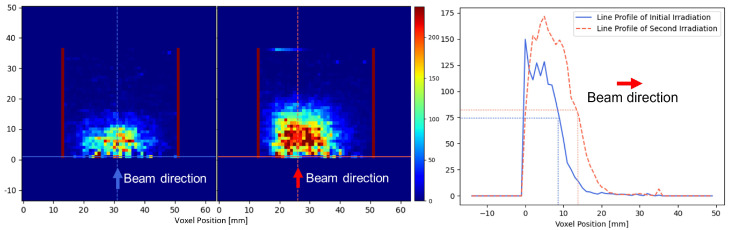
3D localization results in the depth direction. The (**left**) two sub-images (coronal view) show the 2D PET images in a plane containing the identified beam centers of the initial irradiation and the second irradiation, respectively. The (**right**) sub-image shows the intensity profiles along the depth direction for beam centers the two irradiation experiments.

To evaluate the range shift caused by the reduced thickness of the upstream solid water slab, we plotted two line profiles based on the identified beam center locations, as shown in Figure 8. For each profile, we calculated the depth at which the intensity dropped to 50% of the maximum value, yielding 8.3 mm and 13.5 mm, respectively for the two irradiation experiments. The measured range difference was hence calculated as 5.2 mm, deviating by 0.8 mm from the expected value of 6.0 mm. This discrepancy may be caused by image noise. Due to the ultra-high dose rate of the FLASH beam, the reconstructed PET image exhibited higher background noise compared to conventional proton beams, primarily due to elevated prompt-gamma radiation and secondary emissions generated during irradiation [32]. Additionally, the image reconstruction method and the reconstruction iteration selection can also influence the image quality and result accuracy. Despite these factors, the 0.8 mm (<1 mm) range error remained clinically acceptable [33,34], indicating that the multi-panel PET system is promising for an accurate range localization.

## 4. Discussion

In this study, we utilized a multi-panel PET system for the first time to capture PET coincident signals during and immediately after the FLASH beam delivery. Compared to prior studies using a two-detector PET system, the multi-panel PET system offered an enhanced geometrical efficiency, and a broader coverage of the imaging area, resulting in more accurate and reliable signal detection. With the capability of high-precision 3D imaging offered by our PET system, we also achieved 3D localization of the FLASH proton beam. The 3D imaging and localization capability is crucial for preclinical and future clinical FLASH proton therapy, where ultra-high dose rates demand precise beam control and accurate radiation target localization.

The multi-panel PET system is highly promising for capturing and probing the PET activities during and immediately after FLASH delivery, which is beneficial for understanding the microscopic radiation process triggered by the FLASH beam, thereby contributing to exploring the mechanisms of FLASH effects. Under FLASH dose rates, the pattern and timing of generated isotopes may differ from those under conventional dose rates. Measuring these signals provides insight into the microscopic processes occurring during FLASH irradiation, such as nuclear interaction patterns, transient oxygen consumption, and radiation-induced chemistry. The PET signals may be correlated with biological outcomes, helping to elucidate the underlying mechanisms that allow FLASH RT to spare normal tissue while maintaining tumor control [16,35,36,37,38].

Imaging time is a critical factor in clinical practice, especially for accurate dose deposition assessment before biological washout. The multi-panel PET system offers faster PET imaging due to improved geometrical efficiency. Typically, for PET imaging in proton irradiation, imaging acquisition is performed immediately after irradiation, within seconds to minutes. In our study, the PET imaging lasted 11 min, producing high-quality PET images with sufficient accuracy for target localization, which we considered reasonable for clinical or preclinical applications. The prolonged imaging time can lead to a significant reduction in signal strength due to isotope decay and biological washout effects. Additionally, subject movement during PET imaging may severely impact image quality and localization accuracy, particularly for tumors in respiratory-related regions such as the lung and abdomen. Therefore, an optimal imaging time should be carefully evaluated to ensure a balance between imaging time and quality, ultimately enhancing its feasibility for future clinical and preclinical applications.

Given the promising performance of the multi-panel PET system in detecting coincidences and achieving high-precision 3D localization, the recorded data can be effectively leveraged to monitor irradiated tissues during FLASH RT. Moreover, this information may be used to adaptively adjust the treatment plan after each fraction in fractionated FLASH regimens. Although most existing FLASH studies employed a single high-dose treatment, recent research indicated that fractionated FLASH RT regimens can produce similar FLASH effects [39]. For example, Charles et al. implemented a standard-of-care FLASH RT fractionation regimen consisting of 10 fractions (3 Gy per fraction) in mice and evaluated its impact on synaptic plasticity [40]. Their results demonstrated that FLASH RT preserved long-term potentiation, a key measure of synaptic plasticity, whereas conventional RT impaired it. Bourhis et al. delivered whole-brain FLASH irradiation in the striatum of nude mice with a single dose of 10 Gy (1.8 × 10^−6^ s), as well as fractionated doses of 3 × 8 Gy and 5 × 5 Gy with 24-h intervals between fractions [41]. Their results demonstrated the effect of FLASH fractionation schedules on tumor growth delay. Additionally, Mackay et al. suggested that to achieve conformal tumor dosing, a fractionated FLASH regimen with at least three fractions and more than four beam directions is required [2,42]. For these applications, the 3D imaging and localization capabilities of the multi-panel PET system holds great potential for enabling adaptive RT based on imaging feedback.

Our study has several limitations. Most importantly, further advancements in the PET system are desired to improve the capability of signal detection during beam delivery. According to the PET coincidence recording results, the multi-panel PET system demonstrated the capability to handle ultra-high dose rates and rapid isotope decay dynamics, though only to a certain extent. During the initial beam delivery and the immediate post-beam phase, we successfully captured PET signals despite the extremely high photon flux, but with observable issues related to PET dead time and count losses as expected under such conditions. Over the 11 min post-irradiation recording period, the measured decay behavior was consistent with MC simulation results, validating both the production yield and spatial distribution of positron emitters. Nonetheless, a total of $4.1\times 10^{5}$ coincidences were detected, substantially lower than our estimation based on MC simulations. Specifically, for the major reaction channels C12(p,pn)C11, O16(p,pn)O15, and O16(p,2p2n)N13, the yield of ^11^C, ^15^O, and ^13^N per proton at the PET FOV recorded by our MC simulation were $2.2\times 10^{-3}$, $5.1\times 10^{-4}$, and $9.2\times 10^{-5}$. This led to the number of PENs produced by the $3.1\times 10^{10}$ protons

$$N_{11_{C}}\approx 6.7\times 10^{7},\;N_{15_{O}}\approx 1.6\times 10^{7},\;N_{13_{N}}\approx 2.8\times 10^{6}.$$

Using the half-lives of these PENs, the numbers of decays in the first 11 min were

$$D_{11_{C}}\approx 2.1\times 10^{7},\;D_{15_{O}}\approx 1.6\times 10^{7},\;D_{13_{N}}\approx 1.5\times 10^{6}.$$

For the PET system with panel-to-center distance radius r∼8 cm, panel size a=3.3 cm, and n=12 panels, the fractional solid angle of this system with respect to the decay events can be estimated as

(2) $$f_{\mathrm{geom}}=\frac{n}{\pi }\;\arctan\;\left(\frac{a^{2}}{4r\sqrt{r^{2}+\frac{a^{2}}{2}}}\right)\approx 0.156.$$

Let $f_{2\gamma }\approx 0.998$ be the two–photon annihilation branch and $\varepsilon_{\gamma }\approx 0.8$ the single–photon detection probability. Then the expected number of measured events

$$N_{\mathrm{measure}}\;\approx \;\sum_{k}D_{k}\;f_{\mathrm{geom}}\;f_{2\gamma }\;\varepsilon_{\gamma }^{2}\approx \;3.8\times 10^{6},$$

where *k* is the index for PENs. This estimated number is an order of magnitude higher than the measured number of $4.1\times 10^{5}$. To further enhance the PET system, improvements are needed in two key areas. First, the geometrical detection efficiency, currently around 15%, should be increased through optimized detector configurations. Second, count losses under high-rate conditions must be minimized by reducing system dead time, increasing data-processing throughput, and implementing more efficient coincidence-handling algorithms.

While our MC simulations provided valuable insights into PEN productions, several limitations should be addressed in future studies. First, the current model did not include temporal structure of the FLASH proton beam, which is essential for accurately reproducing the early activity dynamics. Second, the comparison was made against ideal isotope activity curves rather than simulated PET detector responses. Under FLASH dose-rate conditions, detector effects such as dead time, pulse pile-up, and other high-rate nonlinearities may substantially alter the measured activity. Future work will therefore incorporate full simulations of the PET detection chain and explicitly model isotope production and decay throughout the irradiation and post-irradiation periods.

As an initial step in exploring the use of PET for FLASH proton irradiation, this work was limited to controlled phantom experiments. While such studies provided critical baseline validation and enabled careful evaluation of detector performance, they did not fully replicate the complexities encountered in biological systems. Future investigations should therefore incorporate in vivo experiments to confirm system performance under physiologically relevant conditions. These studies will be essential for assessing the impact of factors such as biological washout and tissue heterogeneity, which can substantially affect the dynamics of PET signals compared with static phantoms. Moreover, extending the methodology to small-animal models will allow investigation of temporal and spatial correlations between FLASH irradiation, PEN generation, and biological response, ultimately paving the way toward clinical translation.

Another limitation of this study was the relatively simplified evaluation of the temporal structure of beam delivery and post-irradiation PET signals. In particular, our analysis did not fully capture the fine dynamics of coincidence events occurring during the irradiation window and in the immediate seconds thereafter. A more detailed characterization of these temporal patterns is crucial for FLASH research, as the interplay between dose rate, PEN generation, and subsequent decay processes may provide unique insights into the underlying radiation physics and chemistry. Advanced time-resolved acquisition strategies, coupled with improved detector dead-time correction and higher temporal resolution, will be important in future studies to better quantify signal evolution during beam delivery and in the critical post-beam interval.

## 5. Conclusions

This study provided the first 3D imaging and localization of PENs generated by the FLASH proton beam using a multi-detector panel PET system. The system demonstrated the capability to capture PET signals during beam delivery, immediately afterward, and throughout the post-irradiation period, enabling accurate coincidence detection. MC simulations further supported the experimental findings, showing good agreement in signal intensity and spatial distribution. Our results indicated that this multi-panel PET system holds strong potential for image-guided FLASH RT, offering precise beam localization in both range and lateral directions. Despite successfully capturing PET signals under the extremely high photon flux, we observed limitations related to PET dead time and associated count losses. Accurate detection during beam delivery remains challenging due to these factors, highlighting the need for further advancements in the PET system, such as increasing the geometrical detection efficiency and reducing system dead time.

## Figures and Tables

**Table 1:** Summary of representative studies on methods for probing physical and biological effects during FLASH irradiation.

Category	Technique/Method	Key Purpose or Measurement	Representative Studies
Dosimetry (Time-resolved measurement)	Ionization chambers, diamond/plastic scintillator detectors	Characterize per-pulse and instantaneous dose rate under FLASH conditions	Marinelli et al. (2023) [6]; Favaudon et al. (2019) [7]
Optical methods	Cherenkov emission imaging	Provide real-time, pulse-to-pulse visualization of dose output in ultra-high dose-rate electron FLASH RT	Rahman et al. (2022) [8]; Ashraf et al. (2020) [9]
Prompt-gamma & secondary-radiation monitoring	Prompt-gamma timing and radiation signatures	Characterize temporal structure and dose-rate pattern of FLASH beams	Casolaro et al. (2022) [10]; Charyyev et al. (2023) [11]
Ultrafast radiolysis/Oxygen dynamics	Computational and experimental optical oximetry	Quantify radiolytic oxygen depletion and oxygen enhancement ratio during FLASH irradiation	Peng et al. (2025) [12]; González-Crespo et al. (2024) [13]; Cao et al. (2021) [14]; Petusseau et al. (2024) [15]
PET-based real-time beam tracking	Positron emission tomography (PET) with positron-emitting nuclei (^11^C, ^15^O, ^13^N, etc.)	Noninvasive verification of beam delivery and correlation of isotope distribution with dose-rate effects	Abouzahr et al. (2023a) [16]; Abouzahr et al. (2023b) [17]

## Data Availability

The raw data supporting the conclusions of this article are available upon request.
